# Supplementary material for: Cell-Free DNA in Plasma and Serum Indicates Disease Severity and Prognosis in Blunt Trauma Patients
Source: Diagnostics (Basel). 2023 Mar 17;13(6):1150. doi: 10.3390/diagnostics13061150 (PMC10047705; doi:10.3390/diagnostics13061150)
Supplement: Supplementary file 1 [file diagnostics-13-01150-s001.zip › diagnostics-2244470-supplementary.pdf]

Supplementary Materials

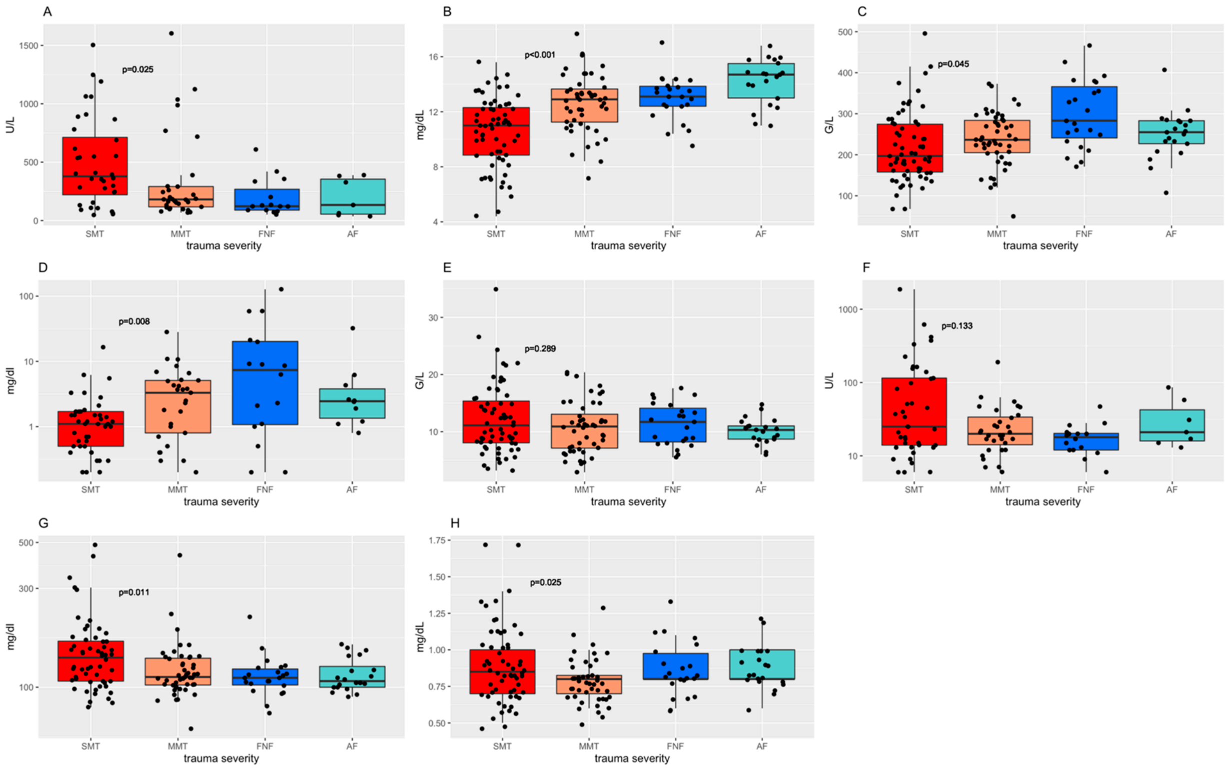

Figure S1. Boxplots of the distribution of investigated blood parameter levels. (**A**) creatinine kinase; (**B**) hemoglobin; (**C**) platelets; (**D**) c-reactive protein; (**E**) leukocytes; (**F**) alanine aminotransferase; (**G**) glucose; (**H**) creatinine. Values for D, F and G were logarithmized for illustration purposes. SMT (severe multiple trauma); MMT (moderate multiple trauma); FNF (femur neck fracture); AF (ankle fracture).

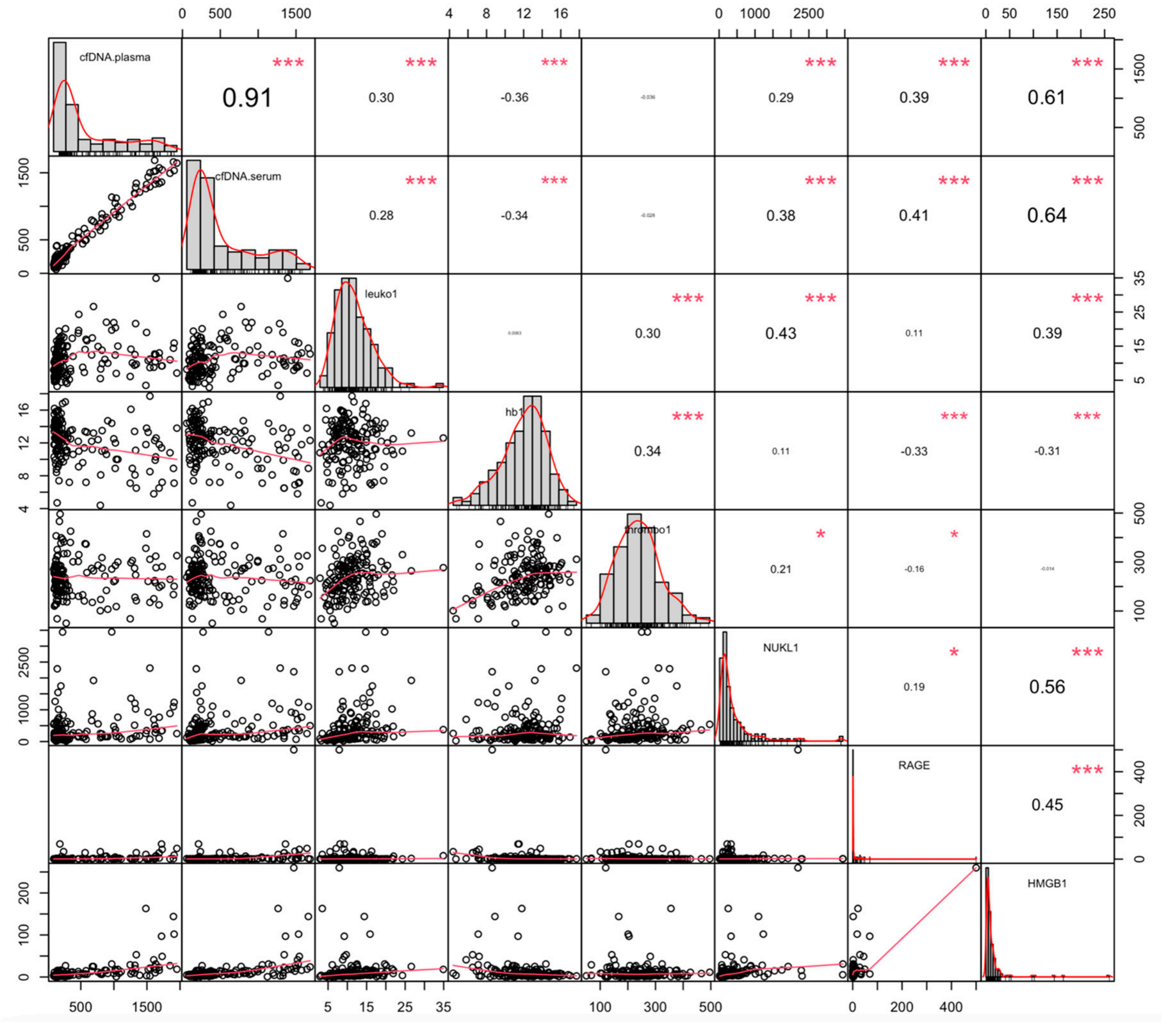

Figure S2. Correlation plots with R values for the additionally investigated blood parameters high-mobility group box 1 (HMGB1), soluble receptor for advanced glycation end products (sRAGE) and nucleosomes (NUKL1), cfDNA in serum and plasma and hematology parameters. Leukocytes (leuko1); hemoglobin (hb1); platelets (thrombo1). * ($p \leq 0.05$); *** ($p \leq 0.001$).
